# Skin Metabolites Define a New Paradigm in the Localization of Skin Tropic Memory T Cells

**DOI:** 10.4049/jimmunol.1402961

**Published:** 2015-05-22

**Authors:** Michelle L. McCully, Paul J. Collins, Timothy R. Hughes, Christopher P. Thomas, Jaak Billen, Valerie B. O’Donnell, Bernhard Moser

**Affiliations:** * Institute of Infection and Immunity, Cardiff University School of Medicine, Cardiff CF14 4XN, Wales, United Kingdom; and; † Department of Laboratory Medicine, Leuven University Hospital, 3000 Leuven, Belgium

## Abstract

The localization of memory T cells to human skin is essential for long-term immune surveillance and the maintenance of barrier integrity. The expression of CCR8 during naive T cell activation is controlled by skin-specific factors derived from epidermal keratinocytes and not by resident dendritic cells. In this study, we show that the CCR8-inducing factors are heat stable and protease resistant and include the vitamin D_3_ metabolite 1α,25-dihydroxyvitamin D_3_ and PGE_2_. The effect of either metabolite alone on CCR8 expression was weak, whereas their combination resulted in robust CCR8 expression. Elevation of intracellular cAMP was essential because PGE_2_ could be substituted with the adenylyl cyclase agonist forskolin, and CCR8 expression was sensitive to protein kinase A inhibition. For effective induction, exposure of naive T cells to these epidermal factors needed to occur either prior to or during T cell activation even though CCR8 was only detected 4–5 d later in proliferating T cells. The importance of tissue environments in maintaining cellular immune surveillance networks within distinct healthy tissues provides a paradigm shift in adaptive immunity. Epidermal-derived vitamin D_3_ metabolites and PGs provide an essential cue for the localization of CCR8^+^ immune surveillance T cells within healthy human skin.

## Introduction

The localization of memory T cells to distinct, nonoverlapping peripheral tissues requires the coordinated expression of specific adhesion molecules and chemokine receptors (1, 2). However, the mechanisms underlying the induction of these specific tissue-homing programs are only beginning to be elucidated. Once these mechanisms are identified, the expression of such factors can be targeted to either promote (vaccination) or dampen (autoimmunity) immune responses at specific tissue sites.

Recent studies have implicated vitamins A and D in the control of T cell homing to the small intestine and skin tissue, respectively (3, 4). Vitamin A is highly concentrated in the gut (5), and retinoic acid, an active metabolite of vitamin A, has been shown to play a crucial role in the induction of the “gut-homing” receptors CCR9 and α4β7 in murine and human T cells (6–8). Conversely, vitamin D_3_, which is produced in the skin in response to UV exposure (9), has been implicated in the regulation of a “skin-homing” mechanism because its active metabolite, 1α,25-dihydroxyvitamin D_3_ (1,25(OH)_2_D_3_), was shown to induce expression of the chemokine receptor CCR10 in human T cells (10). However, the conditions required to induce CCR10 expression did not correlate with induction of other skin-homing receptors, including the adhesion molecule cutaneous lymphocyte–associated Ag, and for naive T cells, the effect was dependent on the presence of IL-12.

We recently reported that the chemokine receptor CCR8 is highly expressed by memory T cells localized in healthy human skin and a small fraction of CLA^+^ memory T cells in blood (11, 12). Further investigation revealed that the induction of CCR8 expression during in vitro T cell activation depended on the addition of soluble skin factors that were produced by epidermal tissue (12). Moreover, cultured keratinocytes but not dermal fibroblasts or skin-unrelated epithelial cell lines produced CCR8-inducing factors, emphasizing the skin selectivity of the CCR8 induction process. Because the epidermis-derived factors responsible for the observed CCR8 induction in T cells were not known, we undertook a detailed investigation into the nature of these factors and their mode of action during T cell activation. In this study, we report that the active vitamin D_3_ metabolite 1,25(OH)_2_D_3_ and PGE_2_ work in concert to induce CCR8 expression in human T cells and that these factors need to be present at the beginning of culture during in vitro T cell activation. Murine skin also produces CCR8-inducing factors, and CCR8-expressing cells are also enriched in mouse skin tissue, indicating that the CCR8-controlled localization of skin-specific memory T cells underlies a conserved mechanism and emphasizes the importance of the skin tissue environment in the homeostasis of the local memory T cell compartment.

## Materials and Methods

### Media and reagents

Complete RPMI (cRPMI) medium consisted of RPMI 1640 plus 2 mM l-glutamine, 1% nonessential amino acids, 1% sodium pyruvate, 50 μg/ml penicillin/streptomycin, 20 mM HEPES, and 10% FBS (Life Technologies). AB-RPMI consisted of cRPMI supplemented with 10% pooled human AB serum. Human T-Activator CD3/CD28 Dynabeads and CFSE were purchased from Life Technologies. Purified anti-mouse CD3 (145-2C11) and CD28 (37.51) Abs and recombinant mouse IL-2 were obtained from BioLegend. Recombinant human IL-12 and IFN-γ were purchased from PeproTech; TNF-α and IL-6 were from Miltenyi Biotech, whereas IFN-α was purchased from Roche. 1,25(OH)_2_D_3_, 25-hydroxyvitamin D_3_, and PGE_2_ were purchased from Sigma-Aldrich. Forskolin, 19R-OH-PGE_1_, CAY10598, Butaprost, L-161,982, AH6809, and SC19220 were purchased from Cayman Chemical. The cAMP-dependent protein kinase A (PKA) inhibitor peptide (PKI)_14__–22_ was obtained from Tocris Bioscience, whereas Raf1 kinase inhibitor 1 and wortmannin were from Enzo Life Sciences. 2-Cl-8-MA-cAMP, N6-MBC-cAMP, and 8-Piperidino-cAMP were purchased from BioLog.

### Human cell isolation and culture

All research involving work with human blood and tissue samples were approved by the local Research Ethics Commission. Informed consent was obtained from each participating subject before sampling in accordance with the Declaration of Helsinki. PBMCs were isolated from healthy donors by density gradient centrifugation using Lymphoprep (Axis-Shield), according to the local ethical guidelines on experimentation with human samples. T cell subsets were purified by MACS, according to the manufacturer’s instructions (Miltenyi Biotec). Purified human T cells were isolated by negative selection using Pan T Cell Isolation Kit II. Naive T cells were further isolated by depleting CD45RO^+^ cells using CD45RO microbeads. Purity was evaluated by flow cytometry and ranged between 92 and 99%.

### Mouse tissue cell isolation

Male and female C57BL/6 Thy1.1^+^ (B6) mice were bred in-house and used between 6 and 12 wk of age. All studies complied with institutional guidelines and the U.K. Home Office for Laboratory Animal Care regulations. Peripheral blood was harvested by cardiac puncture into heparinized tubes and subsequently layered on Lymphoprep to obtain mononuclear cells. Thymus, lymph nodes, and spleen were harvested and mashed through a 40-μm nylon cell strainer to prepare single-cell suspensions. Skin tissue was excised after hair removal, scraped to remove s.c. fat, cut into strips, and floated with the dermis side down in Dispase II buffer at 37°C. After 90 min, the epidermis was separated from dermis, and the epidermal sheets were floated on cRPMI to generate epidermis-conditioned medium (ECM). Alternatively, the whole skin was chopped and incubated in 1× PBS supplemented with 25 μg/ml Liberase TM and 250 μg/ml DNase I (Roche) at 37°C shaking for 30 min. Single-cell suspensions were subjected to Lymphoprep separation prior to staining for flow cytometry or sorted based on CD45 expression using CD45 microbeads (Miltenyi Biotec). Purified naive splenic T cells were isolated by negative selection using the Easysep Mouse Pan Naive T Cell Isolation Kit (Stemcell Technologies), according to manufacturer’s instructions.

### Epidermis-conditioned medium

Freshly isolated epidermal sheets were cultured on AB-RPMI for 3 d. After 3 d, supernatants were harvested, centrifuged, 0.22 μM filtered and either used directly or stored frozen until use. Keratinocyte-conditioned medium (KCM) was generated as described previously (12). To inactivate proteins, conditioned medium was subsequently heated (80°C) or heated and digested with either 0.2–10 U/ml trypsin-agarose beads or proteinase K–Eupergit beads (20 U/ml) for 6 h at 37°C (Sigma-Aldrich). Effectiveness of digestion was determined based on loss of HRP enzymatic activity in spiked medium. For the preparation of mouse skin conditioned medium, ears were split into dorsal and ventral halves and floated dermis side down on cRPMI for 3 d at 37°C. For ECM, dorsal epidermal sheets were prepared and incubated as described previously. Cell-free supernatant was then collected, filtered through a 0.22-μm filter, and stored until use.

### T cell stimulations

Naive T cells (1 × 10^5^ cells/well) were stimulated with anti-CD3/CD28 Dynabeads at a ratio of 4:1 in cRPMI in the presence of various additives for 5 d. For prostaglandin E (EP) receptor inhibition or signaling inhibitor assays, cells were preincubated with inhibitors for 30 min prior to the addition of ECM or PGE_2_ and left overnight. The following day, cells were washed and stimulated with anti-CD3/CD28 Dynabeads at a ratio of 4:1 in cRPMI. For priming experiments, cells were incubated with 100 nM 25(OH)D_3_ or 1,25(OH)_2_D_3_ with or without IL-12 (12.5 ng/ml) overnight and then washed and stimulated with anti-CD3/CD28 Dynabeads in the presence of PGE_2_ or EP receptor agonists.

Murine naive T cells were stimulated with plate-bound anti-CD3 (1 μg/ml) plus soluble anti-CD28 (1 μg/ml) and 30 U/ml recombinant mouse IL-2 in cRPMI supplemented with 50 μM 2-ME for 5 d either alone or in the presence of conditioned medium generated from whole ear skin or epidermal sheets from dorsal skin.

### Flow cytometry

Cells were acquired using a FACSCanto II (BD Biosciences) and analyzed with FlowJo software (Tree Star). The following anti-human mAbs were used: anti-CD3 (UCHT1), anti-CD4 (SK3), anti-CD8 (SK1), anti-CD45RA (HI100), anti-CD45RO (UCHL1), and anti-CCR7 (3D12) from BD Biosciences; and anti-CXCR3 (49801) and anti-CCR10 (314305) from R&D Systems. The following anti-mouse mAbs were used: anti-CD3 (145-2C11), anti-CD4 (RM4-4), anti-CD8 (53-6.7), anti-CD11b (M1/70), anti-CD11c (HL3), anti-CD27 (LG.3A10), anti-CD44 (IM7), anti-CD45 (30-F11), and anti-CD62L (MEL-14). Human CCR8 expression was detected as reported previously (12). Mouse CCR8 expression was detected using a custom-made AF647-labeled murine CCL1 (AF647-muCCL1; Almac). Appropriate isotype controls were included in all cases. Live cells were gated based on their light scatter properties, the exclusion of doublets on forward scatter area/height plots, and the “dumping” of dead cells using Aqua Live/Dead staining (Life Technologies).

### RNA isolation and quantitative PCR

Total RNA was extracted with TRIzol reagent (Invitrogen Life Technologies), according to the manufacturer’s instructions. Genomic DNA was removed by treatment with recombinant DNase I (Ambion Life Technologies), and RNA quality and quantity were determined with a nanodrop spectrophotometer. cDNA was synthesized from 200 ng RNA using the High Capacity RNA-to-cDNA kit from Applied Biosystems. For quantitative RT-PCR, the CCR8 mRNA level was determined using TaqMan Gene Expression assays and TaqMan Universal Master Mix II (Applied Biosystems), according to the manufacturer’s instructions. Reactions were performed using a ViiA7 real-time PCR system (Life Technologies) and were run in triplicate. Water (no template control) and mRNA without reverse transcriptase were used as negative controls. Relative quantification of CCR8 mRNA levels was performed using the comparative Ct method with β-actin as the reference gene and the equation 2^−ΔΔCt^.

### Intracellular cAMP detection

Intracellular cAMP levels were determined using the cAMP Biotrak EIA kit (GE Life Sciences). Briefly, naive T cell were incubated for 30 min in either media alone or media plus 15% ECM, then washed, and lysed using the supplied buffer. The amount of intracellular cAMP present in each sample was determined based on the standard curve and calculated as the fold increase in ECM-treated cultures over media alone.

### PGE_2_ level determination by mass spectrometry

For ECM lipid extraction, a modified Bligh and Dyer extraction was performed. An internal standard of PGE_2_-d_4_ (10 ng) was added to account for variances in sample extraction. A 1-ml sample of ECM was added to 3.75 ml chloroform:methanol (1:2) in a glass extraction vial (13). The mixture was vortexed thoroughly for 1 min before the addition of 1.25 ml chloroform and vortexing for an additional minute. HPLC-grade water (1.25 ml) was added, and the solution was vortexed thoroughly for 1 min. The sample was centrifuged at 500 × *g* for 5 min at 20–22°C before the lower layer was carefully removed with a glass Pasteur pipette and transferred to a clean, labeled glass vial. The samples were dried using a RapidVap or Dryblock with a nitrogen stream at a temperature of 30°C.

Lipids were then analyzed using a C18 Spherisorb ODS2, 5 μm, 150 × 4.6-mm column (Waters, Hertfordshire, U.K.). The mobile phase was composed of 0.1% formic acid in water (solvent A) and 0.1% formic acid in acetonitrile (solvent B), with flow rate 1 ml.min-1. Solvent B was increased from 20 to 42.5% over 50 min, then increased to 90% over 10.5 min, held for 4 min, and then returned to 20% over 1 min. Equilibration time between runs was 14 min. Mass spectroscopy was performed using an ABSciex 4000 Q-Trap, using DP-55V, CE-26V monitoring the parent to daughter *m/z* of 351–271 (14).

### Statistics

Significance testing was performed using the Mann–Whitney *U* test, Dunn’s Multiple Comparison Test, one-way or two-way ANOVA with Dunnett posttest in GraphPad Prism (GraphPad Software). A difference between groups was considered significant when *p* < 0.05.

## Results

### Imprinting of CCR8 expression occurs early during T cell activation

**FIGURE 1.**
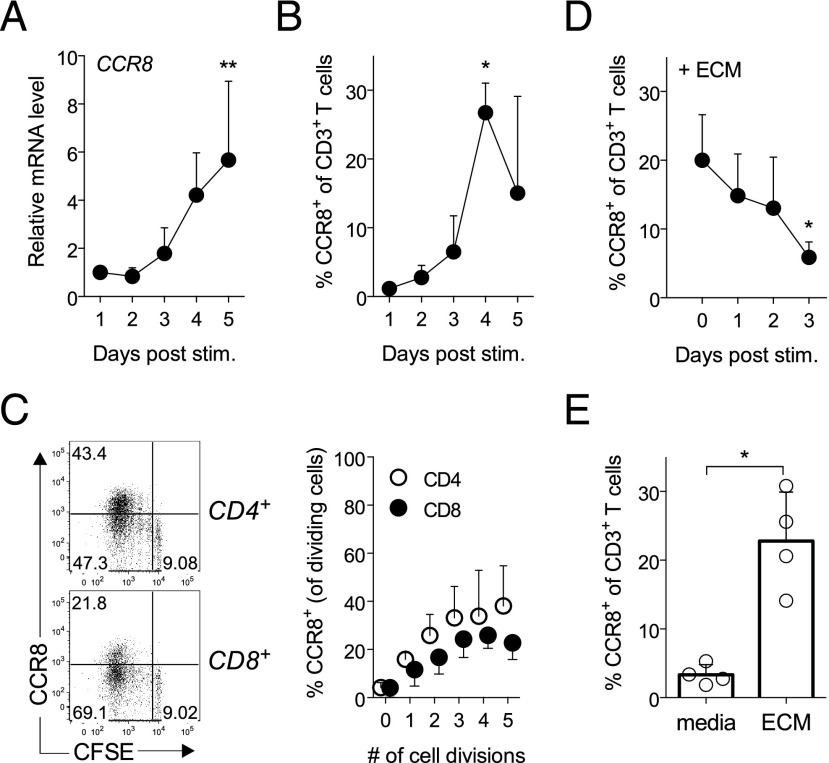
CCR8 expression is imprinted early in activated T cells by skin-derived factors. Naive T cells were stimulated with anti-CD3/CD28 beads in the presence of 15% ECM, and CCR8 expression was determined examined on each day at the RNA level by quantitative PCR (**A**) and at the protein level by flow cytometry (**B**). (**C**) T cell proliferation was monitored in these cultures by CFSE dilution. Representative dot plots of CCR8 expression on CFSE-labeled CD4^+^ and CD8^+^ naive T cells stimulated for 4 d in the presence of ECM are shown. The percentage of proliferating CD4^+^ and CD8^+^ cells expressing CCR8 for each cell division on day 4 is plotted as mean ± SD from three experiments. (**D**) Percentage of activated T cells expressing CCR8 after 5 d in culture when ECM is added either together with anti-CD3/CD28 beads (day 0) or on days 1, 2, or 3 poststimulation. (**E**) Naive T cells were pretreated with ECM for 18 h then washed prior to stimulation with αCD3/CD28 beads. The percentage of T cells expressing CCR8 was assessed after 5 d in culture. Results are plotted as mean ± SD from two to four independent experiments. *p < 0.05, **p < 0.01.

We previously reported that human epidermal keratinocytes release soluble factors that synergize with TCR-activating signals to induce CCR8 expression by naive T cells (12), but the kinetics of their action is not known. First, we determined at which time point CCR8 expression was induced by analyzing RNA and protein expression in activated naive T cells treated with ECM. We found that CCR8 mRNA and protein expression began to rise after 3 d of culture (Fig. 1A, 1B) coinciding with entry into cell division (data not shown). CCR8 was not detected on undivided T cells, irrespective of the length of culture but reached peak expression by day 4–5 on T cells that had undergone the most cell divisions (Fig. 1C).

To determine when the CCR8-inducing factors were most active in relation to TCR triggering, ECM was added either simultaneously with anti-CD3/CD28 beads (day 0) or on days 1, 2, or 3 following treatment with anti-CD3/CD28 beads (Fig. 1D). We found that these factors are most potent when combined with TCR triggering and that sensitivity to the skin-derived factors is lost as the cells begin to divide (day 3). This suggests that the early signaling events mediated by ECM factors and TCR stimuli imprint a homing phenotype that only becomes apparent after 4–5 d in culture (Fig. 1B, 1C); reminiscent of the intricate link between cell proliferation and differentiation as a result of epigenetic alterations (15). Remarkably, even pulsing naive T cells with ECM (i.e., 18-h culture followed by a washing step) prior to TCR triggering resulted in significant CCR8 induction, indicating that the active factor(s) could “imprint” resting T cells for skin homing (Fig. 1E). Therefore, for the effective imprinting of a skin-homing phenotype, exposure of T cells to the CCR8-inducing factor(s) needs to occur either before or during T cell priming. As reported previously, the addition of ECM to naive T cells did not affect their survival (Supplemental Fig. 1A) or proliferation during short-term culture (Supplemental Fig. 1B).

### Intracellular cAMP is an important regulator of CCR8 expression

**FIGURE 2.**
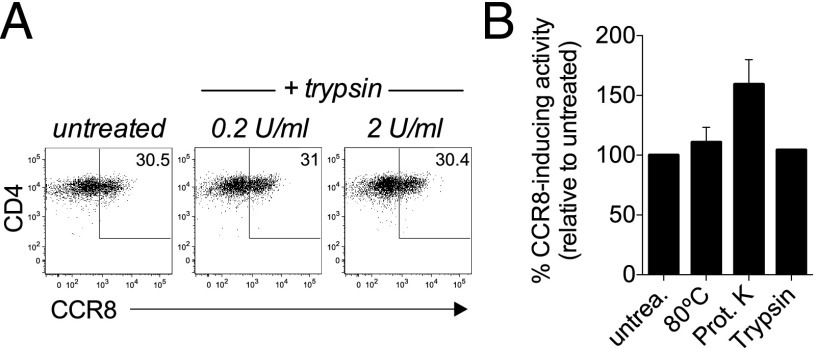
CCR8 induction is mediated by conserved, nonproteinaceous factors. Naive T cells were stimulated for 5 d with anti-CD3/CD28 beads in the presence of ECM or KCM that had been heated to 80°C or digested with trypsin or proteinase K and analyzed for CCR8 expression by flow cytometry after 5 d in culture. (**A**) Representative dot plots are shown for gated CD4^+^ T cells, whereas in (**B**) the CCR8-inducing activity of treated ECM/KCM relative to untreated for CD3^+^ T cells is plotted as mean ± SD from five independent experiments.

We next sought to identify the soluble factor(s) in ECM that are responsible for this activity. Interestingly, the ability to induce CCR8 expression was maintained in ECM or KCM cultures that had been heat treated (80°C) or digested with either proteinase K or trypsin (Fig. 2A, 2B), whereas the enzymatic activity of HRP was abolished under these conditions (data not shown). This indicates that proteins and larger peptides are not responsible for inducing CCR8 expression and implicated lipids and other small molecules as primary candidates.

**FIGURE 3.**
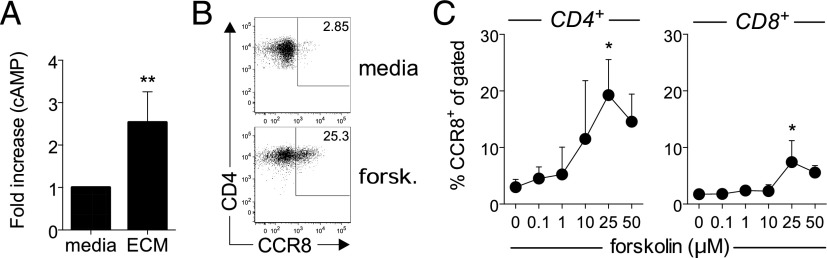
Intracellular cAMP is an important regulator of CCR8 expression. (**A**) Fold increase in intracellular cAMP levels for naive T cells cultured with 15% ECM for 30 min at 37°C compared with media alone from two independent experiments using ECM preps from six healthy skin donors. (**B**) Representative dot plots for CCR8 expression by gated CD4^+^ T cells activated for 5 d in the absence (media) or presence of 10 μM forskolin. (**C**) Percentage of gated CD4^+^ and CD8^+^ T cells expressing CCR8 after 5 d in culture following stimulation with anti-CD3/CD28 beads in the presence of increasing concentrations of forskolin, plotted as mean ± SD from three independent experiments. *p < 0.05, **p < 0.01.

Because many lipids and small molecules act via G protein–coupled receptors (GPCRs), we next tested whether ECM induced the activation of second messengers commonly associated with GPCR-mediated signaling. Incubating naive T cells with ECM for 30 min resulted in a transient 2.5-fold increase of intracellular cAMP levels over media alone (Fig. 3A), suggesting that cAMP-mediated signaling events may be involved in the regulation of CCR8 expression. To test this further, naive T cells were stimulated in the presence of the adenylate cyclase agonist forskolin and found a dose-dependent upregulation of CCR8 expression that peaked at 25 μM (Fig. 3B, 3C), indicating that cAMP is an essential regulator of CCR8 expression. Similar to ECM, forskolin induced a higher percentage of CD4^+^ T cells to express CCR8 than CD8^+^ T cells (Fig. 3C), suggesting that the mechanisms regulating CCR8 expression differ between these two T cell subsets. Importantly, TCR triggering itself induces an elevation in cAMP levels (16) but fails to induce CCR8 expression, suggesting that the effects of ECM and forskolin on cAMP levels are qualitatively/quantitatively different from those derived from the TCR.

**FIGURE 4.**
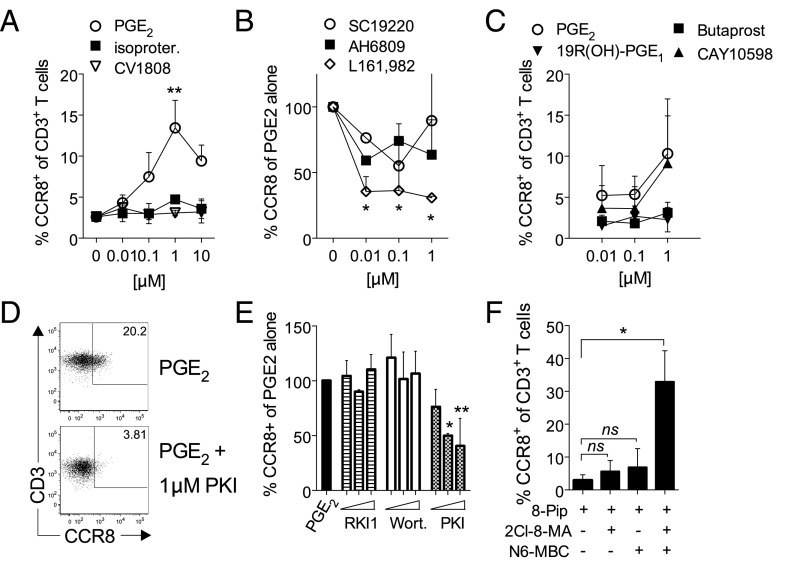
PGE_2_ mediated activation of PKA results in CCR8 expression. (**A**) Naive T cells were stimulated for 5 d with anti-CD3/CD28 beads in the presence of 0.01–10 μM PGE_2_, isoproterenol or CV1808. The percentage of gated CD3^+^ T cells expressing CCR8 was determined by flow cytometry and plotted as mean ± SD from two to six independent experiments. (**B**) Naive T cells were incubated with PGE_2_ alone or PGE_2_ in the presence of 0.01–10 μM of antagonists against EP1 (SC19220), EP1/2 (AH6809), or EP4 (L161,982) prior to stimulation with anti-CD3/CD28 beads. After 5 d, the percentage of cells expressing CCR8 was determined by flow cytometry and plotted as a percentage of cells expressing CCR8 relative to PGE_2_ alone. Results are plotted from two independent experiments. (**C**) Naive T cells were stimulated in the presence of 0.01–1 μM PGE_2_ or the EP-selective agonists 19R(OH)-PGE_1_ (EP1), butaprost (EP2), or CAY10598 (EP4). After 5 d, the percentage of CD3^+^ T cells expressing CCR8 was determined by flow cytometry, and results are plotted as mean ± SD from four independent experiments. (**D** and **E**) Naive T cells were pretreated with 0.01–1 μM of the inhibitors Raf1 kinase inhibitor 1 (RKI1), wortmannin, or PKA inhibitor peptide PKI_14–22_ (PKI) prior to stimulation in the presence of PGE_2_. Representative dot plots for CCR8 expression by gated CD3^+^ T cells stimulated in the presence of PGE_2_ alone or PGE_2_ + 1 μM PKI are shown in (D), whereas bar graphs depicting the percentage of CD3^+^ T cells expressing CCR8 for inhibitor-treated groups relative to PGE_2_ alone are plotted in (E). (**F**) Naive T cells were stimulated for 5 d in the presence of 0.1 mM of site-specific cAMP agonists selective for activation of PKA-I (2-Cl-8-MA-cAMP + 8-Pip-cAMP) and PKA-II (N6-MBC-cAMP + 8-Pip-cAMP) (20). The percentage of CD3^+^ T cells expressing CCR8 was determined by flow cytometry after 5 d and plotted as mean ± SD from two independent experiments. *p < 0.05, **p < 0.01.

We next tested GPCR agonists that are known to activate G_αS_ and found that the addition of PGE_2_, but neither the A_2_ adenosine receptor agonist CV1808 nor the β2 adrenergic receptor agonist isoproterenol, resulted in increased CCR8 expression by activated T cells (Fig. 4A). PGE_2_ exerts its activity through four GPCRs (EP1–4) with EP2 and EP4 being the most highly expressed by naive T cells (17). By incubating naive T cells with EP-specific antagonists, we found that the EP4 receptor–selective antagonist L161,982 inhibited PGE_2_-induced CCR8 expression more consistently and potently than either SC19220 (EP1) or AH6809 (EP1–3) (Fig. 4B), suggesting that EP4 was primarily responsible for regulating CCR8 expression. In support, only the EP4-specific agonist CAY10598 was capable of mimicking the PGE_2_ effect, whereas the EP1/EP3 agonist 19R(OH)-PGE_1_ and the EP2 agonist butaprost failed to do so (Fig. 4C).

Although both EP2 and EP4 activate cAMP signaling, EP4 has also been shown to activate the PI3K signaling pathway (18). As such, we next tested which pathway was primarily responsible for inducing CCR8 expression by preincubating cells with increasing concentrations of either the PKA-selective inhibitory peptide PKI, the PI3K inhibitor wortmannin, or the Raf1 inhibitor Raf1 kinase inhibitor 1 prior to the addition of PGE_2_. For this experiment, the pulsing of naive T cells with PGE_2_ with or without the signaling inhibitors prior to the addition of anti-CD3/CD28 beads was necessary to prevent the signaling inhibitors from interfering with T cell activation. In accordance with our forskolin data (Fig. 3), we found that only incubation with PKI significantly reduced PGE_2_-induced CCR8 expression on activated T cells (Fig. 4D, 4E).

The downstream effects of cAMP are thought to be mediated by the various isoforms of PKA (19). Therefore, we tested whether there was a preference for PKA type I or type II in the regulation of CCR8 expression by comparing the effect of PKA subtype–specific cAMP analogs (20). Interestingly, whereas the preferential activation of either PKA-I or PKA-II failed to induce significant CCR8 expression, combining these analogs resulted in a strong synergy indicating that the activation of both isoforms is essential for CCR8 expression (Fig. 4F). This finding clarifies why neither TCR triggering alone nor activation of the β2 adrenergic receptor are effective at inducing CCR8 expression as these receptors preferentially activate PKA-I (21, 22). We conclude that the cAMP elevating agents PGE_2_ and forskolin are potent CCR8-inducing agents when added at micromolar concentrations.

### Active vitamin D_3_ synergizes with PGE_2_ to induce CCR8 expression

**FIGURE 5.**
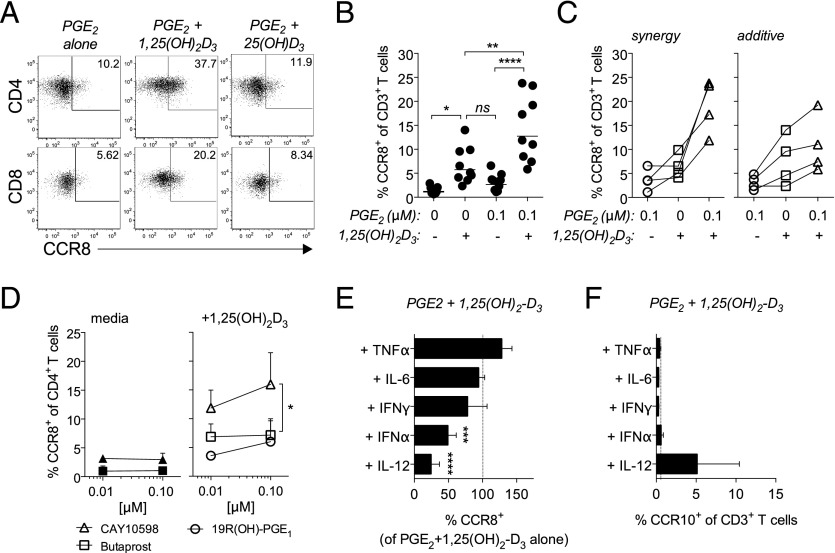
Ability of 1,25(OH)_2_D_3_ to enhance PGE_2_-mediated CCR8 induction. (**A**–**C**) Naive T cells were stimulated in the presence of 0.1 μM PGE_2_ alone, 0.1 μM 1,25(OH)_2_D_3_ alone, or PGE_2_ in combination with either 0.1 μM 1,25(OH)_2_D_3_ or 0.1 μM 25(OH)D_3_. (A) Representative dot plots for CCR8 expression on gated CD4^+^ and CD8^+^ T cells after 5 d are shown, whereas in (B) the percentage of gated CD3^+^ T cells, expressing CCR8 is plotted for each responding donor (*n* = 9). Line (**—**) denotes geometric mean. (C) The response of each donor to these treatments is plotted according to whether the resulting percentage of CCR8 expressing T cells in response to PGE_2_ + 1,25(OH)_2_D_3_ exceeded the sum of either metabolite alone (synergy; *left panel*) or whether it was equal to the sum of the responses induced by each metabolite alone (additive; *right panel*). (**D**) Naive T cells were stimulated in the presence of various EP receptor agonists at 0.01 or 0.1 μM in media alone or in combination with 0.1 μM 1,25(OH)_2_D_3_. The percentage of CD4^+^ T cells expressing CCR8 was determined after 5 d and plotted as mean ± SD from two independent experiments. (**E** and **F**) Naive T cells were stimulated in the presence of 0.1 μM PGE_2_ + 0.1 μM 1,25(OH)_2_D_3_ in the absence or presence of 12.5 ng/ml IL-12, 100 U/ml IFN-α, 12.5 ng/ml IFN-γ, 50 ng/ml IL-6, or 20 ng/ml TNF-α. After 5 d, the percentage of CD3^+^ T cells expressing CCR8 (E) or CCR10 (F) was determined by flow cytometry. The percentage of cells expressing CCR8 is plotted in (E) as mean ± SD relative to T cells treated with PGE_2_ + 0.1 μM 1,25(OH)_2_D_3_ alone from five independent experiments. *p < 0.05, **p < 0.01, ***p < 0.001, ****p < 0.0001.

PGE_2_ alone is not as effective as ECM at inducing CCR8 expression, and PGE_2_ levels in ECM are below micromole concentrations (44.8 ± 33.9 nM; 14.95–83.72 nM; *n* = 5), suggesting that other factor(s) may work in concert with PGE_2_. Previous reports highlight the critical role for vitamins A and D in the regulation of tissue-homing receptor expression (3). However, neither of these vitamins alone showed significant activity in our previous human CCR8 induction experiments. To our surprise, we found that the combination of 1,25(OH)_2_D_3_, a metabolite typically found in ECM cultures, with suboptimal levels (0.1 μM) of PGE_2_ resulted in a significant increase in the percentage of CD4^+^ and CD8^+^ T cells expressing CCR8 over PGE_2_ alone (*p* < 0.0001) or 1,25(OH)_2_D_3_ alone (*p* < 0.01; Fig. 5A, 5B). Interestingly, combining these metabolites resulted in a synergistic increase in CCR8 expression for five of the nine responding T cell donors tested, whereas the remaining four donors only showed an additive response (Fig. 5C). The reason for this difference is unclear but may reflect donor variability in the responsiveness to these metabolites as well as the timing of delivery. However, it is important to note that for some donors the response to 1,25(OH)_2_D_3_ alone was relatively high when compared with the median (Fig. 5B) or when compared with previously published experiments (12). For these donors, it is conceivable that the effect of 1,25(OH)_2_D_3_ alone precluded any synergistic effect when combined with PGE_2_. Of note, and in line with our findings with ECM, CCR8 expression in CD4^+^ T cells routinely exceeded the levels observed in CD8^+^ T cells (data not shown). This effect was specific for the active metabolite of vitamin D_3_ because neither the precursor 25(OH)D_3_ nor the vitamin A metabolite, all-trans-retinoic acid, showed synergistic activity (Fig. 5A; data not shown). This synergistic effect could also be seen when 1,25(OH)_2_D_3_ was combined with suboptimal levels of the EP4 receptor agonist CAY10598 (Fig. 5D) as well as forskolin (data not shown) but not the EP1/EP3 agonist 19R(OH)-PGE_1_ or the EP2 agonist butaprost (Fig. 5D).

We previously reported that the addition of IL-12 to T cell–keratinocyte cocultures inhibited the keratinocyte-mediated CCR8 induction (12). In agreement, in this paper, we report that the addition of IL-12 inhibited the synergy between 1,25(OH)_2_D_3_ and 0.1 μM PGE_2_ during induction of CCR8 expression (Fig. 5E), which is in contrast to CCR10, whose modest expression depended on this cytokine (Fig. 5F). Likewise, the addition of IFN-α also significantly reduced the percentage of activated T cells expressing CCR8 after treatment with PGE_2_ plus 1,25(OH)_2_D_3_, whereas IFN-γ, IL-6, and TNF-α did not have this effect. The inhibitory effect of IL-12 and IFN-α could be seen for both CD4^+^ and CD8^+^ T cells (Supplemental Fig. 2A), and similar data were obtained when using only 50 nM PGE_2_ in combination with 1,25(OH)_2_D_3_ (Supplemental Fig. 2B) or when T cells were stimulated in the presence of ECM (Supplemental Fig. 2C).

**FIGURE 6.**
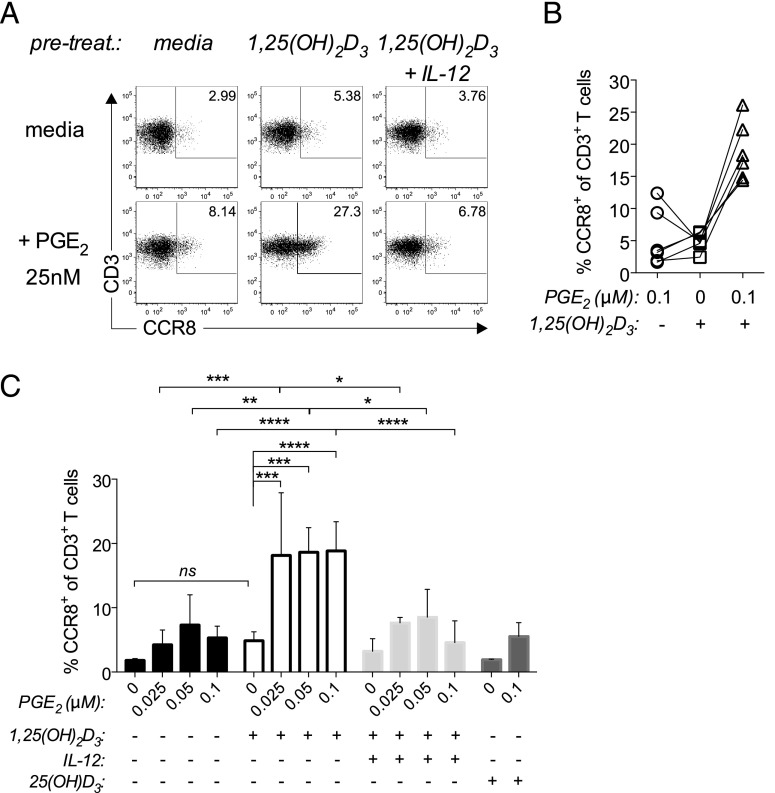
1,25(OH)_2_D_3_ primes PGE_2_-mediated CCR8 induction. Naive T cells were left untreated or pulsed with either 1,25(OH)_2_D_3_ alone, 1,25(OH)_2_D_3_ + IL-12, or 25(OH)D_3_ for 18 h, washed, and then stimulated with anti-CD3/CD28 beads in the absence or presence of suboptimal concentrations of PGE_2_ (0.025, 0.05, or 0.1 μM). (**A**) Representative dot plots for CCR8 expression on gated CD3^+^ T cells 5 d poststimulation for T cells pretreated overnight with media, 1,25(OH)_2_D_3_ alone, or 1,25(OH)_2_D_3_ + IL-12, followed by stimulation in the absence (media) or presence of 0.025 μM PGE_2_ are shown. (**B**) The percentage of CD3^+^ T cells expressing CCR8 after 5 d for all treatment groups is plotted as mean ± SD from three to six independent experiments. (**C**) The percentage of CD3^+^ T cells expressing CCR8 for T cells stimulated in the presence of 0.1 μM PGE_2_ (i.e., pulsed with media alone), T cells pulsed with 1,25(OH)_2_D_3_ alone, followed by stimulation in the absence of PGE_2_ or T cells pulsed with 1,25(OH)_2_D_3_, followed by stimulation in the presence of PGE_2_ is plotted for each donor (*n* = 6). The effect on CCR8 expression of pulsing T cells with 1,25(OH)_2_D_3_, followed by stimulation in the presence of PGE_2_ exceeds the sum of the effects of either metabolite alone. *p < 0.05, **p < 0.01, ***p < 0.001, ****p < 0.0001.

Because ECM possessed the ability to “imprint” a skin-homing phenotype in responding T cells (Fig. 1E), we next examined whether 1,25(OH)_2_D_3_ could prime naive T cells for CCR8 expression. To do so, naive T cells were first pulsed with either 1,25(OH)_2_D_3_ alone, 1,25(OH)_2_D_3_ plus IL-12, or its precursor 25(OH)D_3_ for 18hrs, washed and then stimulated with anti-CD3/CD28 beads in the presence of a suboptimal concentrations of PGE_2_ (0.025–0.1 μM). Consistent with our findings with ECM, the pulsing of T cells with 1,25(OH)_2_D_3_, but not its inactive precursor, 25(OH)D_3_, resulted in a significant increase in CCR8 expression over either PGE_2_ alone (*p* < 0.001 for 0.025 μM PGE_2_) or 1,25(OH)_2_D_3_ alone (*p* < 0.001; Fig. 6A, 6B), indicating that skin-derived 1,25(OH)_2_D_3_ does not need to be present at the time of activation and plays an important role in conditioning T cells for skin homing. Interestingly, and in contrast to data shown in Fig. 5C, pulsing naive T cells with 1,25(OH)_2_D_3_ prior to activation in the presence of PGE_2_ resulted in a synergistic response for all donors tested (Fig. 6C), whereas pulsing with 1,25(OH)_2_D_3_ in the presence of IL-12 inhibited this synergy, revealing an early inhibitory effect of IL-12. These findings are in line with the view that the active metabolite of vitamin D_3_ acts on local T cells before they interact with local DCs or migratory DCs in the draining lymph nodes following their exit from the skin.

### Regulation of CCR8 expression by skin tissue is conserved between mice and humans

**FIGURE 7.**
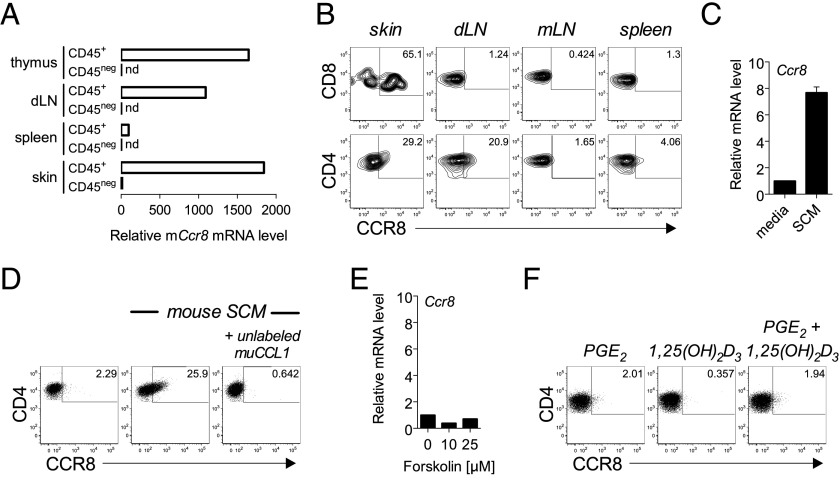
Conserved regulation of murine CCR8 expression by skin tissue. (**A** and **B**) Expression of murine CCR8 was evaluated by quantitative PCR in purified CD45^+^ and CD45^neg^ cell populations isolated from the skin, spleen, skin-draining lymph nodes, and thymus (A) and by flow cytometry on gated CD8^+^ and CD4^+^ memory (CD44^hi^) T cells isolated from mouse skin, skin-draining LN (dLN), mesenteric LN (mLN), and spleen (B) from wild-type B6 mice stained with AF647-muCCL1. Data in (A) are plotted relative to the skin CD45-negative population. (**C** and **D**) Naive splenic T cells were stimulated in vitro with plate-bound anti-CD3 plus soluble anti-CD28 in the absence or presence of mouse skin–conditioned medium (SCM). After 5 d, cells were harvested and assayed for CCR8 expression at the RNA level by quantitative PCR (C) or stained with AF647-muCCL1 ± excess unlabeled muCCL1 (D) to detect surface CCR8 expression by flow cytometry on gated CD4^+^ T cells. Results in (C) are plotted as mean ± SD from two independent experiments (*n* = 2–3 mice) and dot plots in (D) are representative of four independent experiments from cells pooled from two to three mice each. (**E**) Naive splenic T cells were stimulated in vitro with plate-bound anti-CD3 plus soluble anti-CD28 in the absence or presence of 10 or 25 μM forskolin. After 5 d, cells were harvested and assayed for CCR8 expression at the RNA level by quantitative PCR. (**F**) Naive splenic T cells were stimulated in vitro with plate-bound anti-CD3 plus soluble anti-CD28 in the presence of 1 μM PGE_2_, 0.1 μM 1,25(OH)_2_D_3_ alone, or PGE_2_ + 1,25(OH)_2_D_3_. After 5 d, cells analyzed for CCR8 expression by flow cytometry. nd, not detected.

A recent study suggested that CCR8 expression is also restricted to skin tissue in mice (23). As a result, we next examined tissues from healthy B6 mice and found that CCR8 mRNA expression is largely restricted to the CD45^+^ fraction of immune cells localized in the skin and skin-draining lymph nodes, and consistent with a previous report, CCR8 mRNA was also detected at high levels in murine thymocytes (Fig. 7A) (24, 25). Further examination using custom-made AF647-labeled murine CCL1 (AF647-muCCL1) determined that, as in humans, CCR8 is primarily expressed by memory (CD44^hi^) T cells present in the skin and skin-draining lymph nodes (Fig. 7B) as well as by some single-positive and double-negative thymocytes (Supplemental Fig. 3). In murine skin, expression was detected on both CD4^+^ and CD8^+^ skin T cells, whereas expression in draining lymph nodes was restricted to the CD4^+^ subset, consistent with previous reports showing that the recirculation patterns of the two memory T cell subsets differ (26–28). In contrast, significant levels of CCR8 were not detected on lymphocytes isolated from the spleen, mesenteric lymph nodes, or even peripheral blood (Fig. 7B, Supplemental Fig. 3).

On the basis of the comparatively conserved expression patterns of CCR8, we next asked whether the mechanism regulating human CCR8 expression would also regulate murine CCR8 expression. Activation of naive mouse T cells in the presence of mouse skin–conditioned medium resulted in the upregulation of murine CCR8 mRNA (Fig. 7C) and surface protein expression (Fig. 7D) signifying a conserved cross-species regulation of CCR8 expression by skin tissue. As for induction of human CCR8, this effect was more prominent on murine CD4^+^ than CD8^+^ T cells and could be replicated using ECM prepared from dorsal epidermal sheets (data not shown). However, unlike human T cells, the elevation of intracellular cAMP was not required for murine CCR8 expression as neither forskolin (Fig. 7E) nor cAMP analogs (data not shown) were capable of inducing CCR8 expression at the mRNA (Fig. 7E) or protein level (data not shown) in activated mouse T cells. In agreement, the addition of either PGE_2_ alone or PGE_2_ plus 1,25(OH)_2_D_3_ were also ineffective in inducing CCR8 expression (Fig. 7F). Inactivity of these factors may reflect differences in human and mouse T cell responsiveness to 1,25(OH)_2_D_3_ as was reported in the regulation of CCR10 expression (10). Of note, vitamin D synthesis is absent in murine skin (29), explaining why this metabolite cannot participate in immune modulatory processes in the skin of mice and possibly other nocturnal vertebrates. However, the possibility that other factors combine with 1,25(OH)_2_D_3_ in the regulation of mouse CCR8 cannot be excluded. Nevertheless, it is clear that CCR8-expressing memory T cells accumulate in the skin of mice and humans and that skin tissue-derived factors regulate CCR8 expression in both species and, as such, underlies a conserved mechanism in the localization of skin-specific memory T cells.

## Discussion

Control of tissue-resident memory T cells by distinct tissue environments represents a new paradigm in peripheral immune surveillance. The importance of this paradigm is underscored by the fact that healthy peripheral tissues harbor vast numbers of memory T cells whose presence is essential for maintaining tissue integrity and longevity. In this study, we show a mechanism for the control of human CCR8 expression involving the skin-produced metabolites 1,25-dihydroxyvitamin D_3_ and PG E_2_. Activation of the cAMP/PKA-signaling pathway was found to be essential for the induction of CCR8, and in agreement, PGE_2_ alone could induce CCR8 expression. However, this effect was only observed at high (micromolar) concentrations of PGE_2_, whereas induction of CCR8 at concentrations of PGE_2_ produced by steady-state epidermis only occurred in the presence of the active vitamin D metabolite, 1,25(OH)_2_D_3_. Our data highlight an important role for vitamin D_3_ in modulating the expression of skin-homing chemokine receptors in a context-driven manner. In the presence of the inflammatory cytokines IL-12 (10) or TNF-α plus IL-6 (30), 1,25(OH)_2_D_3_, induces low-level expression of the chemokine receptor CCR10 in T cells, whereas in the presence of basal levels of PGE_2_, we found that 1,25(OH)_2_D_3_ synergistically enhanced expression of CCR8. Importantly, we also found that the addition of the type 1 proinflammatory cytokines IL-12 and IFN-α inhibited CCR8 expression, suggesting that CCR8 and CCR10 are induced under different circumstances, although further investigation is needed. In support, we have reported that CCR8 is expressed by a majority of memory T cells isolated from healthy human skin, whereas CCR10 is largely absent (12).

Skin is the primary site for vitamin D_3_ synthesis because its precursor 7-dehydrocholesterol (or provitamin D) is uniquely produced at high levels in this tissue (31). Upon exposure to sunlight, this precursor is converted to previtamin D_3_, which then isomerizes spontaneously over time to vitamin D_3_. This conversion occurs almost exclusively within the epidermis because UVB penetration is limited to this layer (31). Although the majority of vitamin D_3_ is then transported to the liver and kidney for conversion to 25(OH)D_3_ and its active form 1,25(OH)_2_D_3_, respectively, basal keratinocytes also express the necessary enzymes (25-hydroxylases and 1-hydroxylase) to convert vitamin D_3_ to 1,25(OH)_2_D_3_, indicating that skin tissue itself is a source of the active metabolite (32). Local production is essential as 1,25(OH)_2_D_3_ is required for keratinocyte differentiation (33) and skin tissue defense (34), but the levels found in circulation are low (<100 pM) and are unlikely to sufficiently supply the epidermis. Interestingly, the photosynthesis of vitamin D appears to be an adaptation of diurnal vertebrates to sunlight because nocturnal animals, including rodents, do not possess this ability (29, 35). This provides an explanation for why certain skin tissue–expressed immune-associated genes such as cathelicidin (36), CCR10 (10, 37), and CCR8, as shown in this study, are positively regulated by 1,25(OH)_2_D_3_ in humans but not in mice.

Vitamin D_3_ plays an important immune-modulatory role because deficiency in this vitamin in humans is associated with increased incidence of infection and autoimmunity (38). In terms of T cell differentiation, 1,25(OH)_2_D_3_ is thought to play a predominant inhibitory role because it indirectly promotes the differentiation of regulatory T cell subsets through its actions on dendritic cells (DCs) (39, 40), whereas it primarily inhibits the differentiation of other Th cell subsets (41). Whether the effects of 1,25(OH)_2_D_3_ on T cell differentiation are due solely to indirect actions via DCs is unclear, but our findings along with those from Sigmundsdottir et al. (10) clearly show that 1,25(OH)_2_D_3_ acts directly on human T cells to promote skin homing receptor expression. On the basis of these data, it is possible that the decreased production of vitamin D_3_ in aged skin could be a factor in the age-related decline in the local skin immune memory response (42, 43).

PGE_2_ has also been previously implicated in the regulation of skin immune cell migration. Specifically, PGE_2_ was shown to promote the maturation and migration of Langerhans cells, and as in our study, this effect was dependent on signaling through EP4 (44). More recently, PGE_2_ was shown to indirectly promote a skin-homing phenotype in responding T cells as a result of its action on DCs. A study by Stock et al. (45) demonstrated that skin-produced PGE_2_ was responsible for inhibiting the expression of retinoic acid–producing enzymes by DCs. As a consequence, T cells primed with these “skin-conditioned” DCs had higher expression of P-selectin ligands and accumulated in murine skin tissue (45). However, it remains unclear as to how interference with vitamin A metabolism in DCs promotes skin tropism in activated T cells. In human T cells, retinoid acid, the active form of vitamin A, did not interfere with CCR8 expression (12).

Like vitamin D_3_, PGs are also important for skin tissue homeostasis (46). In fact, PGE_2_ is one of the main cutaneous eicosanoids produced by both epidermal keratinocytes and dermal fibroblasts. However, only primary cultures of epidermal keratinocytes are capable of producing both PGE_2_ and 1,25(OH)_2_D_3_, consistent with their unique ability to support CCR8 expression (12). Many of the effects of PGE_2_ on immune cells have been attributed to the activation and accumulation of intracellular cAMP and its downstream activation of cAMP-dependent PKA. In agreement, in this paper, we document that the PGE_2_-mediated induction of CCR8 expression in naive T cells required PKA signaling. Although inhibitory at high levels (47, 48), the generation of intracellular cAMP has also been shown to be necessary in the differentiation of Th1 and Th17 cells (49) and also to promote the responsiveness of primed T cells to restimulation (50).

Our study emphasizes the unique role played by tissues in maintaining the tissue-specific immune surveillance T cell repertoire. We propose that the skin tissue environment provides signal 3 in the multistep process of immune memory T cell formation. TCR triggering during adhesive interaction of naive T cells with Ag-presenting DCs provides signal 1 and leads to primed T cells receptive for costimulatory signals (signal 2) that drive T cell differentiation and expansion. Epidermis-derived factors, including 1,25(OH)_2_D_3_ and PGE_2_, as shown in this study, play an ambassador role in relaying the tissue-homing information (signal 3) to local, emigrating, and/or skin-draining lymph node T cells. Because CCR8^+^ T cells predominate in healthy skin, we suggest that signal 3 is primarily active during the steady state (i.e., under conditions of immune homeostasis). This suggests that signal 3 could occur prior to T cell activation (signal 1 and 2), consistent with our priming data. Under inflammatory conditions, the effect of skin factors may be overridden by inflammatory mediators (i.e., IL-12 and IFN-α) that ensure the recruitment of newly generated effector T cells in response to inflammatory chemokines. We do not yet know how CCR8^+^ T cells relate to inflammatory T cells and, indeed, when during the course of primary immune responses CCR8^+^ T cells are being formed. We have noted that excessive TCR triggering (i.e., high anti-CD3 concentrations neutralized the effect of CCR8-inducing factors [unpublished observations]), which agrees with the view that CCR8 becomes expressed during the resolution phase of inflammation when the Ag density on DCs becomes limited. A recent study examining the differentiation of skin-resident memory T cells in mice found that CCR8 mRNA levels increased in tissue-resident T cells during the resolution phase of an immune response (>7 d postchallenge) (23). It remains to be investigated whether CCR8 expression occurs locally (i.e., at the late stage of inflammation) or in tissue-specific lymph nodes that drain the necessary signal 3 tissue factors. Even though the CCR8-inducing factors appear to differ between mice and humans, our findings show that the localization of CCR8^+^ memory T cells and the regulation of CCR8 expression by skin tissue are conserved, indicating that it is now possible to investigate the kinetics of CCR8 expression in mouse models of skin infections and vaccination.

## Supplementary Material

Data Supplement
