# Supplementary material for: Skin Metabolites Define a New Paradigm in the Localization of Skin Tropic Memory T Cells
Source: J Immunol. 2015 May 22;195(1):96–104. doi: 10.4049/jimmunol.1402961 (PMC4472944; doi:10.4049/jimmunol.1402961)
Supplement: Data Supplement [file JI_1402961.zip › JI_1402961_Supplemental_Figures_1.pdf]

Supplemental Data:

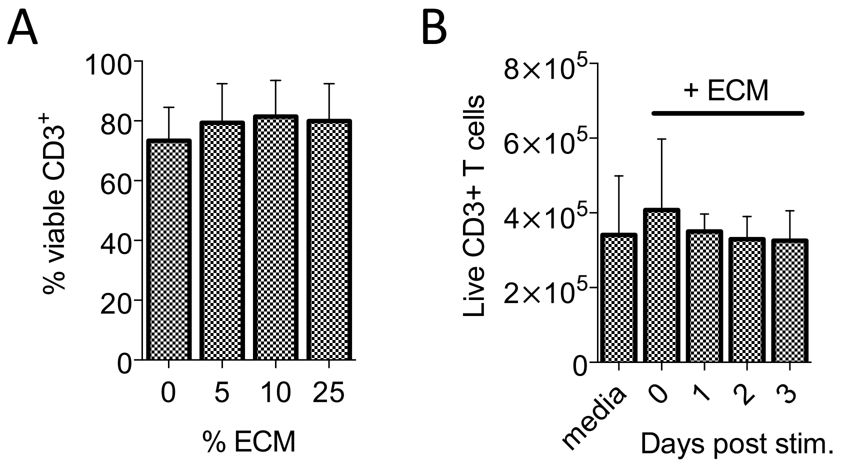

Figure S1. ECM does not alter the viability or proliferative capacity of activated naïve T cells. (A) The percentage of viable $CD3^+$ T cells recovered after 5 days in culture with αCD3/CD28 beads in the absence (0%) or presence of increasing amounts of ECM. **(B)** The total number of live $CD3^+$ T cells recovered after activation with αCD3/CD28 beads alone (media) or when ECM is added either together with αCD3/CD28 beads (day 0) or on days 1, 2, or 3 post stimulation.

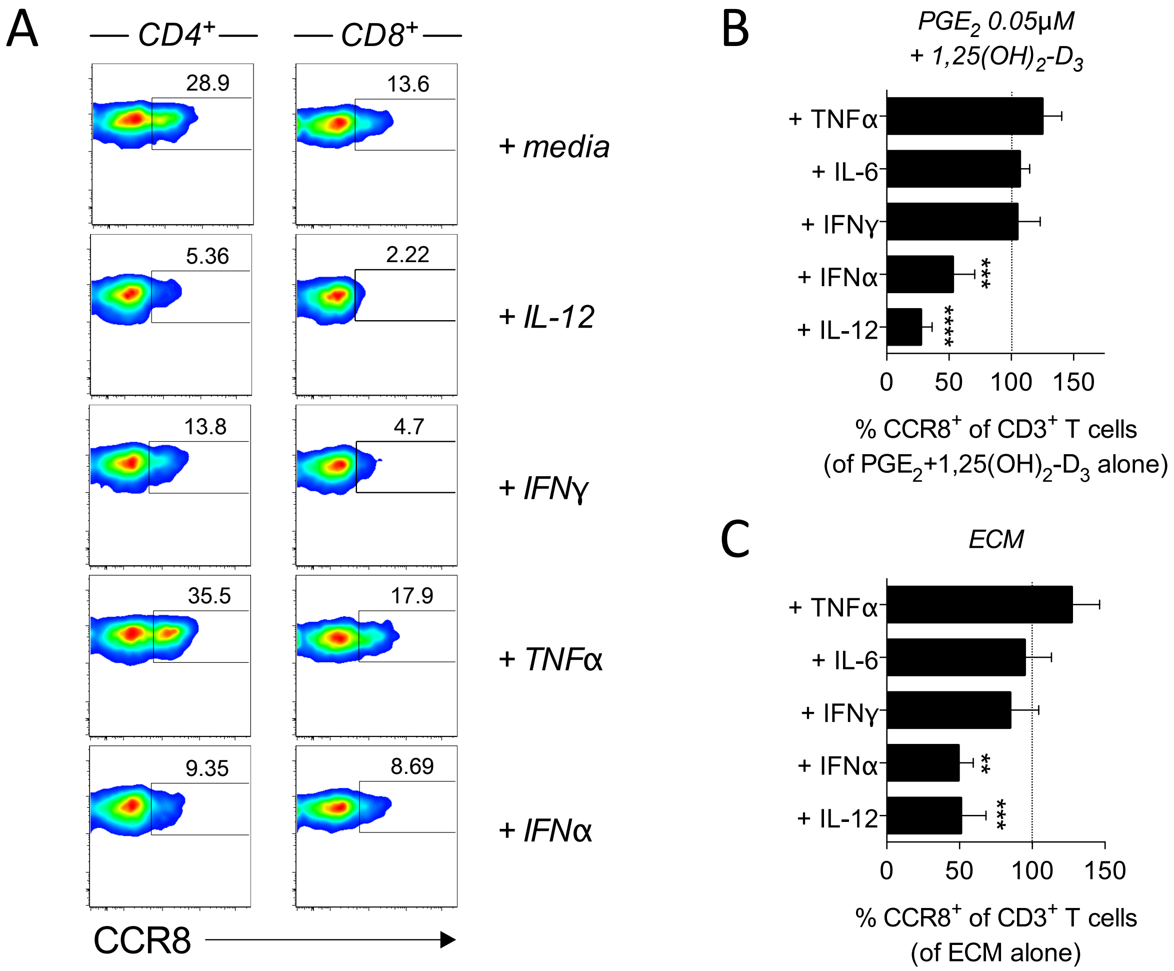

Figure S2. Type 1 pro-inflammatory mediators inhibit ECM and PGE2+1,25(OH)2D3-mediated CCR8 induction. Naïve T cells were stimulated for 5 days with αCD3/CD28 beads in the presence of either 0.05 µM PGE_2 + 0.1 µM $1{,}25(OH)_2D_3$ **(A & B)** or 20% ECM **(C)** alone or in combination with 12.5 ng/ml IL-12, 12.5 ng/ml IFNγ, 20 ng/ml TNFα, 50 ng/ml IL-6 or 100 U/ml IFNα. **(A)** Representative flow cytometric dot plots for CCR8 expression by gated $CD4^+$ and $CD8^+$ T cells stimulated in the presence of PGE_2 + $1{,}25(OH)_2D_3$ alone (+ media) or in combination with the various cytokines. **(B & C)** The percentage of $CD3^+$ T cells expressing CCR8 was determined after 5 days and plotted as mean ± SD for the percentage relative to control T cells treated with PGE2 + $1{,}25(OH)_2D_3$ alone (B) or ECM alone (C) from 4-5 independent experiments. $^{**}P<0.01$, $^{***}P<0.001$, $^{****}P<0.0001$.

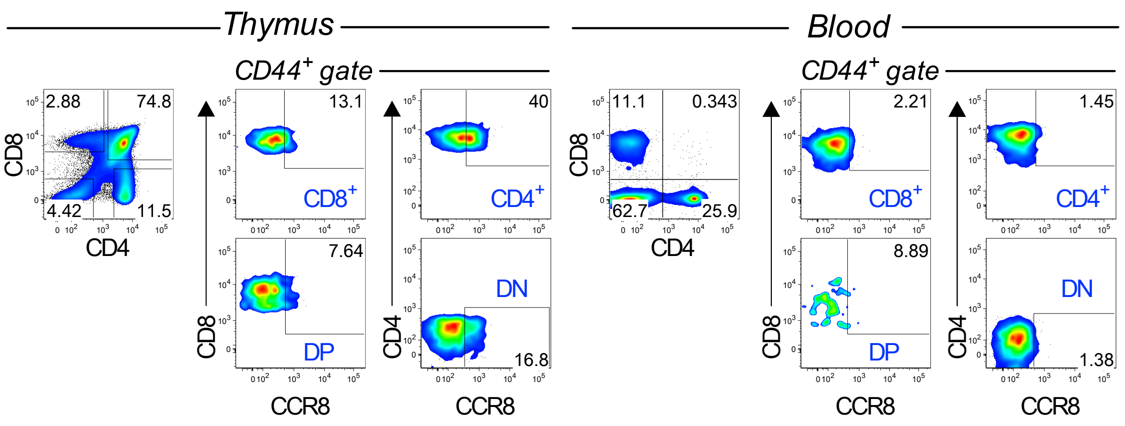

Figure S3. CCR8 is expressed by mouse thymocytes. Expression of murine CCR8 was evaluated by flow cytometry on gated CD44$^+$ T cells from the CD8$^+$, CD4$^+$, CD4$^+$CD8$^+$ (double positive; DP) or CD4$^-$CD8$^-$ (double negative; DN) populations isolated from the thymus and peripheral blood of wild-type B6 mice stained with AF647-muCCL1.
